# Impact of a Training Program on Oncology Nurses’ Confidence in the Provision of Self-Management Support and 5As Behavioral Counseling Skills

**DOI:** 10.3390/cancers15061811

**Published:** 2023-03-16

**Authors:** Doris Howell, Patrick McGowan, Denise Bryant-Lukosius, Ryan Kirkby, Melanie Powis, Diana Sherifali, Vishal Kukreti, Sara Rask, Monica K. Krzyzanowska

**Affiliations:** 1 Princess Margaret Cancer Research Institute, University of Toronto, Toronto, ON M5G 2M9, Canada; 2 School of Public Health & Social Policy, University of Victoria, Victoria, BC V8P 5C2, Canada; 3 School of Nursing, McMaster University, Hamilton, ON L8S 4L8, Canada; 4 Northern Ontario School of Medicine, Sudbury, ON P3E 2C6, Canada; 5 Medical Oncology Division, Princess Margaret Cancer Centre, University Health Network, Toronto, ON M5G 2C4, Canada; 6 Department of Medicine, University of Toronto, Toronto, ON M5S 1A1, Canada; 7 Medical Oncology Division, Royal Victoria Hospital, Barrie, ON L4M 6M2, Canada

**Keywords:** cancer, self-management support, coaching, training, nurses, pre/postsurvey

## Abstract

**Simple Summary:**

Cancer patients and their families require support to effectively self-manage the medical, emotional, and lifestyle consequences of cancer. In this paper, we describe a training program that showed improvement in oncology nurses confidence in the microskills required for the provision of self-management support to patients for application in routine care and in roles as cancer coaches before and after training at three cancer centres in Ontario, Canada. Self-management support is lacking in cancer care and little attention has been focused on the required preparation of nurses to provide self-management support and behavior change counseling. Our training program may have potential for improving nurses’ provision of self-management support, however, further testing in a larger population of nurses is required to assess effects on nurses skills and its impact on patient uptake of self-management behaviors and health outcomes.

**Abstract:**

Background: Cancer patients and their families play a central role in the self-management of the medical, emotional, and lifestyle consequences of cancer. Nurses with training in self-management support can enable cancer patients to better manage the effects of cancer and treatment. Methods: As part of a randomized controlled trial, we developed a training program to build nurses’ confidence in the provision of self-management support (SMS). The SMS skills taught were adapted from the Stanford Peer Support training programs and embedded within the 5As (Assess, Advise, Agree, Assist, and Arrange) behavioral counseling process. We evaluated the impact of the training program on oncology nurses’ and coaches’ confidence using a Student’s t-test for paired samples in a nonrandomized, one-group pre/postsurvey. Results: Participants were experienced oncology nurses from three participating cancer centers. A two-tailed Student’s t-test for paired samples showed a significant improvement in nurses’ confidence for the 15 SMS microskills targeted in the training between the pretest and post-test as follows: for Center 1, a mean difference of 0.79 (t = 7.18, *p* ≤ 0.00001); for Center 2, a mean difference of 0.73 (t = 8.4, *p* ≤ 0.00001); for Center 3, a mean difference of 1.57 (t = 11.45, *p* ≤ 0.00001); and for coaches, a mean difference of 0.52 (t = 7.6, *p* ≤ 0.00001). Conclusions: Our training program improved oncology staff nurses’ and cancer coaches’ confidence in 15 SMS microskills and has potential for SMS training of nurses in routine care.

## 1. Background

Globally, there is increasing recognition of the central role of cancer patients and caregivers in achieving better health outcomes through effective self-management of the acute and chronic effects of cancer [1,2]. Self-management involves the application of a set of cognitive and behavioral skills to manage the medical aspects of cancer, including physical symptoms and treatment effects, psychosocial consequences, role and lifestyle changes [3,4,5], and other tasks for living with cancer [6]. Individuals with cancer require the knowledge, skills, and confidence to tailor their daily behaviors to manage dynamic disease and symptom fluctuations, reduce complications and late-effect risks, and optimize well-being and survival [7,8].

Cancer self-management can be daunting and may not be in the individuals’ usual repertoire of disease self-management skills or health behaviors [9], and they may require self-management support (SMS) to build their capacity and self-efficacy for effective disease self-management [10]. Healthcare providers who are skilled in the systematic provision of SMS and in coaching patients on the use of health behaviors and core skills such as goal setting, action planning, and problem-solving [11] can empower and enable patients in the self-management of cancer and health [12].

Oncology nurses, as one of the largest service provider groups in cancer care, can play pivotal roles in the provision of SMS in routine care and as cancer coaches [13,14]. Systematic reviews have shown that SMS, delivered by nurses educated and skilled in facilitating patient activation in self-management, can result in positive behavioral change and better clinical outcomes in typical chronic conditions (e.g., reduced blood pressure in hypertension and lower A1c in diabetes) [15,16]. SMS also improves physical symptoms (e.g., fatigue), anxiety, and quality of life in cancer populations [17,18,19]. However, oncology nurses require specific knowledge and skills in the provision of SMS and coaching for behavior change and positive attitudes toward collaborative engagement of patients as partners in care [20]. SMS involves more than just condition-related education: it also involves coaching of patients in the cognitive and behavioral application of self-management behaviors to address specific problems (e.g., cancer fatigue) and the core skills inclusive of goal setting and taking action, system navigation, implementing problem-solving strategies, communicating with healthcare professionals, self-monitoring, decision making, resource utilization and self-tailoring of health behaviors to optimize health during and after cancer treatment [21].

The purpose of this article is to describe the development and impact of a SMS training program on oncology nurses’ confidence in the 15 microskills necessary for delivering effective SMS in routine care and acting as cancer coaches. The training program was developed in the context of a randomized controlled trial entitled the Self-Management and Activation to Reduce Treatment Toxicities (SMARTCare) that targeted patient management of acute treatment toxicities (NCT03849950). Ethics approval for the study was obtained from the Ontario Clinical Oncology Group, Hamilton, ON. Canada.

## 2. Materials and Methods

**Figure 1:**
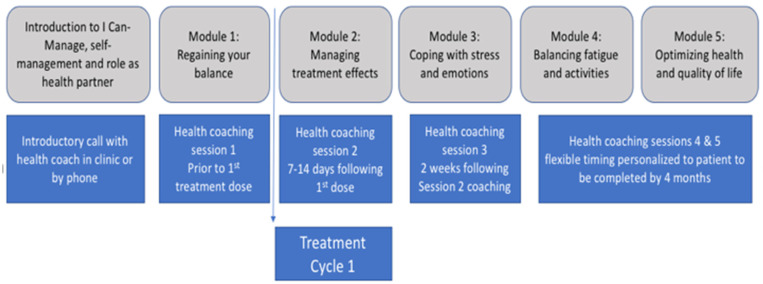
SMARTCare intervention timed across the treatment trajectory.

Briefly, the SMARTCare intervention comprised two components: an online self-management education program for patients (I-Can Manage.ca) and telephone-based, nurse-delivered cancer coaching targeting patients diagnosed with lung cancer, colorectal cancer, and lymphoma, who were scheduled to receive first-line or metastatic parenteral or oral treatment. Study participants were randomized to receive the online program and 5 sessions of cancer coaching (approximately 45 min per session) timed for completion across the treatment trajectory or a usual care control condition (Figure 1). Oncology clinic nurses were trained in practical skills of SMS (e.g., teach back) that could be applied in routine practice [22]. Oncology clinic nurses and two nurses from each disease site were purposively selected as cancer coach intervention nurses from ambulatory clinics across 3 regional cancer centers in Ontario, Canada, to participate in the training program. The results of the trial suggest promising results for nurse-delivered SMS coaching for improving patient activation [23].

We adapted an existing telephone-based, peer-led SMS program based on the Stanford approach with demonstrated effectiveness in diabetes [24] to cancer populations. The training was provided by experts in SMS and coaching (PM and DH). All oncology clinic nurses received 4 h of training in the fundamentals of SMS that targeted the application of specific strategies to support patients’ application of core self-management skills for the management of cancer treatment toxicities and health promotion (smoking cessation, physical activity, and healthy eating).

**Figure 2:**
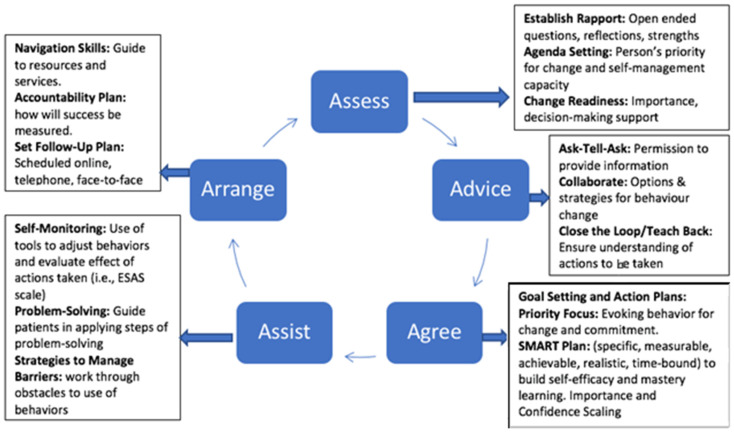
Self-management support microskills integrated in 5As behavior counseling process; ESAS = Edmonton Symptom Assessment System. Used as routine symptom screen in centers.

SMS techniques and microskills taught in the training program were embedded within the teaching of the 5As (Assess, Advise, Agree, Assist, and Arrange) process for counseling patients in the use of self-management behaviors (Figure 2) [25].

**Figure 3:**
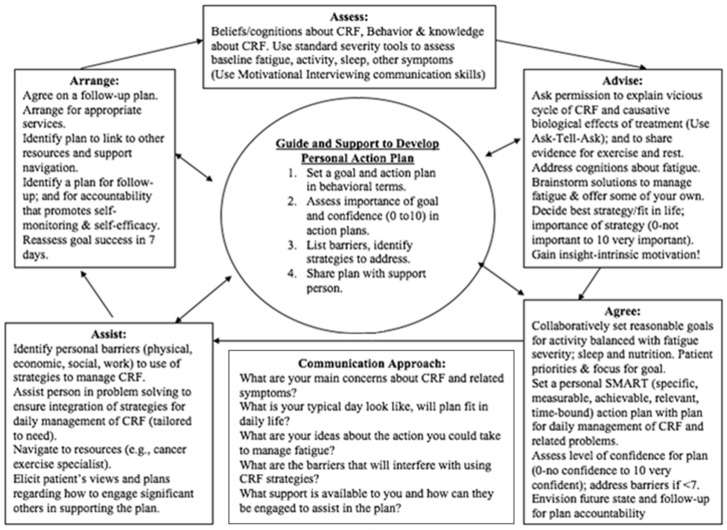
Example of application of 5As to cancer fatigue.

The training emphasized the fundamental microskills of establishing rapport, setting a shared agenda, assessing readiness using rulers (importance and confidence), ask–tell–ask, closing the loop, and teach back for practical application by staff nurses and cancer coaches. This approach is consistent with recommendations of provincial nursing guidelines [26] and based on the Stanford approach for developing peer leaders’ core self-management support skills [24]. The 5As model is recommended for ensuring that healthcare professionals use a sequential set of actions to help individuals to become better self-managers [27]. We also demonstrated the application of the 5As for behavioral counseling of patients in the management of specific treatment toxicities (e.g., fatigue) (Figure 3) and for coaching self-management of treatment toxicities such as immunotherapy (Supplement S1 in Supplementary Materials). The training program was participatory and case-based, and it included skills practiced through modeling and role playing.

**Figure 4:**
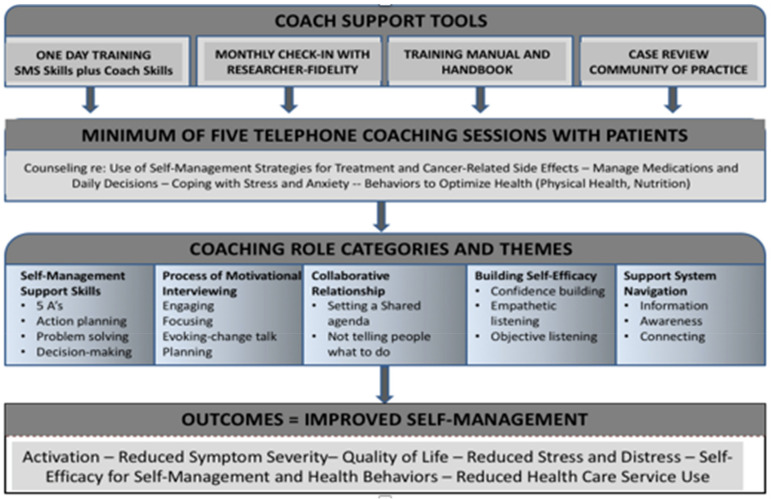
SMARTCare cancer SMS coaching intervention model.

Nurses seconded as cancer coaches to deliver self-management coaching in the intervention arm of the trial participated in the fundamentals of SMS (4 h), then received an additional 7 h of an enhanced training program that targeted deeper consolidation of skills in applying the 5As and advanced SMS skills, including goal setting and action planning, problem solving, decision making, and motivational interviewing OARS communication skills (open-ended questions, affirmations, reflections, and summarization). Cancer coaching was defined as “a person-centred, collaborative, strength-based care process that educates, engages, and motivates patients within a coaching context to enhance self-management capability and self-efficacy in applying problem-specific self-management strategies, core skills, and health behaviors to reduce the immediate and long-term physical and emotional consequences of cancer and cancer treatment based on health coaching models for other chronic conditions” [28]. The SMARTCare intervention and training program was guided by our cancer coaching model adapted from the diabetes peer support training program that shows the range of skills emphasized in the training [24] (Figure 4). This approach uses the patients’ frame of reference (i.e., their experiences, definitions of health and well-being, values, and preferences) as the starting point to identify health goals, and then provides navigation, education, support, practical guidance, and facilitation of uptake of behaviors and skills to help the client manage symptoms and achieve their health goals.

Oncology nurses designated as coaches in the intervention arm were also guided by a manual to standardize the coaching process and support intervention fidelity. The manual consisted of the objectives, content, and processes to be covered in each of the five coaching sessions for the SMARTCare intervention. Recording forms were included for nurses to document each call. Additionally, instruction was provided for making monthly telephone calls and focusing the coaching conversation on four key areas: (1) how patients were managing their cancer and treatment toxicities, medications, home, and daily life; (2) using the problem-solving process; (3) making action plans to support a behavioral goal; and (4) how to access and navigate locate community resources. Cancer coaches also received mentorship through monthly community-of-practice meetings with experienced cancer coaches who were focused on case review and supporting skills consolidation. Cancer coach interactions with a client after the first 2 sessions were observed using a fidelity coaching checklist (Supplement S2 in Supplementary Materials), and coaches were provided with constructive feedback on their application of coaching skills and guidance for further consolidation. 

We developed a purpose-built, pre/postsurvey to evaluate nurses’ confidence using a Likert scale (from 0 (no confidence) to 5 (very confident)) in the application of the 5As and 15 related micro-skills (Supplement S3 in Supplementary Materials). Descriptive statistics were used to describe sample characteristics and mean scores across survey items and nurses’ satisfaction with the training program. We checked the data for normality using Shapiro–Wilk tests, and the normality assumption was met. Thus, a Student’s t-test for paired samples was used to examine mean differences in oncology nurses’ confidence in using the 15 micro-skills for SMS from the pre- to post-test survey for each of the three centers. A *p*-value of 0.05 was considered significant.

## 3. Results

A total of 40 clinic nurses, who were mostly diploma prepared with >10 years cancer experience and certified in oncology, completed the fundamentals of SMS training and 8 nurses received additional training as cancer coaches (Table 1). Most nurses had limited pretraining awareness of the concepts of self-management, self-management support, and patient activation (Table 2).

A statistically significant improvement in the 15 SMS microskills for nurses from the three centers participating in the training program and separately for coaches was observed as follows: for Center 1, an overall mean difference of 0.79 between pre- and postsurvey (t = 7.18, *p* ≤ 0.00001); for Center 2, a mean difference of 0.73 (t = 8.4, *p* ≤ 0.00001); for Center 3, a mean difference of 1.57 (t = 11.45, *p* ≤ 0.00001); and for coaches, a mean difference of 0.52 (t = 7.6, *p* ≤ 0.00001; Table 3).

Overall, the training, facilitation, and methods for teaching (i.e., skills practice) were positively viewed, and the time to practice skills using cases and role playing was highly valued (Table 4).

## 4. Discussion

We developed a SMS training program to be tested in a feasibility RCT, with preliminary effects shown for improvement in patient activation favoring the online self-management education program plus coaching compared with a usual care control group [23]. In this paper, we described our training program and showed positive benefits for improving nurses’ knowledge and self-confidence in the microskills necessary for the provision of self-management support and coaching within the 5As behavior counseling framework. While there are training programs for health coaches, this is one of the few programs that additionally targets training of oncology nurses in self-management support for application within their practices. SMS is essential to the achievement of a more person-centered, psycho-educational approach that can enable patients to live well with cancer as an acute and chronic disease [29]. Self-management education combined with nurse-led SMS is considered integral for enabling patients to effectively manage chronic conditions, including complex illnesses such as cancer [30,31,32]. However, few studies have addressed SMS training for nurses, and this preparation is lacking in basic and graduate nurse education programs [33]. Recently, a core curriculum was developed through international consensus that can be used to guide future undergraduate and graduate education programs [34]. However, less is known about how to build oncology nurses’ application of SMS in routine care, and a gap was identified in the integration of SMS in routine oncology care [35]. We recognize that skills consolidation requires more than a one-time training program. We did not evaluate the application of SMS in routine care by oncology staff nurses, and this should be examined in future research. We did assess the use of skills by cancer coaches and identified that more time and practice in skill consolidation will be essential in future SMS training and that online learning may facilitate the learning process [36]. The practical issues of training healthcare professionals and their integration of self-management skills within their practice will require consideration.

There is an urgent need to understand how a system-wide approach to SMS can become embedded and integrated into routine practice and the essential training components and skill consolidation strategies that are critical for scale and spread. Training in the fundamental skills of SMS could be included as part of new staff orientation and ongoing professional development. However, this will require cancer organizations to set similar expectations for performance for SMS within practice and as a standard of quality care in cancer programs [37].

There is still much to understand regarding the critical elements and how best to build the self-efficacy and capacity of nurses to deliver effective SMS in routine care and as cancer coaches and to ensure their ongoing competence. Further creative multimedia training approaches, such as the use of standardized patients for skills consolidation and rigorous evaluation, are needed to measure the uptake of SMS in the practice repertoire of nurses and other healthcare professionals. Oncology nurses in ambulatory cancer clinics are ideally positioned to deliver SMS that could build cancer patients’ self-efficacy and capacity for self-management of treatment toxicities but will require flexibility in adopting this approach within traditional patient education programs and the time to ensure its delivery in rapid, episodic clinic visits.

This study has limitations, including the self-selection of nurses as cancer coaches, the use of a purpose-built survey for program evaluation, and the one-group design without a control comparator. A power calculation was not performed given the feasibility nature of the work; thus, statistical significance should be interpreted in this context. It is also uncertain if SMS skills will be maintained after the trial without ongoing efforts toward embedding and normalizing SMS in routine care.

## 5. Conclusions

As noted in the Chronic Disease Self-Management Framework, one of the main objectives of health services is to support self-management, which needs to be embedded within a system and includes knowledgeable and confident individuals, prepared clinicians, and a responsive and flexible service delivery structure [38]. This study demonstrated the feasibility of training oncology nurses to deliver SMS coaching. Further research is needed to evaluate the effectiveness of interventions for integrating and sustaining nurse-led SMS into routine care.

## Figures and Tables

**Table 1:** Baseline demographics of ambulatory clinic staff nurses by center and for coaches.

Question	Center 1	Center 2	Center 3	Coaches
	*n* = 11	*n* = 14	*n* = 15	*n* = 8
Years working as a registered nurse, µ (σ)	22.45 (9.88)	29.18 (8.53)	16.8 (11.53)	21.5 (9.24)
Years working in oncology, µ (σ)	17.45 (9.48)	19.46 (8.95)	13.87 (10.13)	18.63 (10.42)
Current area of work, *n* (%) Ambulatory clinics Other	11 1	13 1	14 1	7 1
Highest level of nursing education, *n* (%) * Diploma Bachelor’s Master’s	7 4 -	8 5 1	3 10 2	4 2 2
Completion of professional certifications, *n* (%) * CNA certification in oncology CNA certification in palliative care The de Souza Institute certification Other certifications	10 2 1 2	11 - - 2	12 1 3 2	6 1 2
Previously received professional education/training addressing the principles/practices of SMS, *n* (%) No Yes, within last 2 years Yes, over 2 years ago	8 - 3	10 - 4	11 2 2	6 2

* = participants could select more than one answer.

**Table 2:** Pretraining awareness of concepts of self-management and support.

Concept	Center 1	Center 2	Center 3	Coaches
	*n* = 11	*n* = 14	*n* = 15	*n* = 8
Patient activation, µ (σ)	2.45 (0.93)	3.00 (0.96)	1.73 (1.03)	3.25 (0.71)
Self-management support, µ (σ)	3.55 (0.93)	2.93 (0.92)	2.20 (0.86)	3.50 (0.53)
Self-management, µ (σ)	3.45 (0.93)	3.14 (0.86)	2.33 (0.82)	3.38 (0.52)
Cancer as a chronic condition, µ (σ)	4.09 (0.94)	4.00 (0.78)	3.07 (1.22)	4.00 (0.53)

SMS = self-management support; µ = mean; σ = standard deviation.

**Table 3:** Mean scores for pre/postsurvey confidence in self-management support skills by center and for coaches.

Question			Center 1		Center 2		Center 3		Coaches	
			*n* = 11		*n* = 14	*n* = 16	*n* = 15		*n* = 8	
			Pre	Post	Pre	Post	Pre	Post	Pre	Post
a—Defining SMS, µ (σ)			3.6 (0.97)	4.09 (0.83)	3.21 (1.12)	4.19 (0.75)	2.07 (0.8)	4.53 (0.52)	3.5 (0.76)	4.25 (0.71)
b—Supporting patients in making difficult decisions, µ (σ)			3.82 (0.6)	3.82 (1.08)	4 (0.78)	4.13 (0.72)	2.73 (0.88)	4.20 (0.56)	3.25 (0.71)	3.75 (0.71)
c—Using 5As model in providing SMS, µ (σ)	Assess	Establish rapport	4.09 (0.54)	4.55 (0.69)	4.07 (1)	4.5 (0.63)	3.38 (0.52)	4.47 (0.64)	3.88 (0.83)	4.13 (0.64)
		Setting visit agenda	3.09 (1.04)	4.27 (0.79)	3.57 (1.09)	4.06 (0.68)	4.00 (0.53)	4.20 (0.56)	3.5 (1.07)	4 (0.76)
		Assess client readiness	3.27 (0.9)	4.45 (0.69)	3.5 (1.02)	4.19 (0.66)	2.67 (0.9)	4.33 (0.49)	3.38 (0.74)	3.75 (0.71)
		Goal setting	3.45 (0.82)	4.3 (0.67)	3.43 (1.02)	4.19 (0.66)	2.60 (0.91)	4.33 (0.49)	3.38 (0.74)	3.50 (0.53)
	Advise	Tailoring strategies	2.73 (1.01)	4.18 (0.6)	3.14 (1.17)	4.06 (0.68)	2.13 (0.92)	4.20 (0.68)	2.88 (0.35)	3.63 (0.52)
		Ask–tell–ask	2.64 (0.92)	4.09 (0.54)	3.14 (1)	4.44 (0.63	2.33 (1.05)	4.33 (0.49)	3.00 (0.53)	3.88 (0.83)
		Closing the loop	3.09 (0.83)	4.18 (0.6)	3.00 (1.18)	4.31 (0.7)	2.33 (1.18)	4.47 (0.52)	3.00 (0.53)	4 (0.76)
	Agree	Action plans and follow-up	3.82 (0.6)	4.55 (0.52)	3.43 (1.09)	4.19 (0.66)	2.60 (1.06)	4.27 (0.46)	3.25 (0.89)	4 (0.76)
	Assist	Problem solving	3.91 (0.54)	4.36 (0.5)	3.50 (0.94)	4.31 (0.7)	2.80 (0.86)	4.20 (0.68)	3.25 (0.71)	3.63 (0.74)
		Linking to community resources	4.00 (0.45)	4.27 (0.47)	3.64 (0.84)	4.00 (0.73)	3.00 (0.93)	4.33 (0.49)	3.38 (0.52)	3.63 (0.52)
		Teaching self-monitoring skills	3.55 (0.82)	4.27 (0.47)	3.07 (1.07)	3.94 (0.77)	2.73 (0.8)	4.13 (0.64)	3.25 (0.71)	3.63 (0.92)
		Review goal and action plan	3.36 (0.67)	4.36 (0.5)	3.43 (0.94)	4.19 (0.54)	2.73 (0.7)	4.33 (0.62)	3.25 (0.71)	3.88 (0.64)
	Arrange	Follow-up	3.91 (0.7)	4.5 (0.53)	3.93 (0.92)	4.31 (0.6)	3.07 (0.88)	4.33 (0.62)	3.75 (0.46)	4.00 (0.76)
t-Test Results				Mean Diff 0.79 t = 7.18 *p* ≤ 0.00001		Mean Diff 0.73 t = 8.48 *p* ≤ 0.00001		Mean Diff 1.57 t = 11.45 *p* ≤ 0.00001		Mean Diff 0.52 t = 7.6 <0.00001

SMS = self-management support; µ = mean; σ = standard deviation.

**Table 4:** Study participants’ evaluation of self-management support training by center and for coaches.

Question		Center 1	Center 2	Center 3	Coaches
		*n* = 11	*n* = 16	*n* = 15	*n* = 8
Overall, µ (σ)	Valuable use of time	3.10 (0.99)	4.00 (0.63)	4.47 (0.64)	4.38 (0.52)
	Relevant to professional practice	4.00 (0.89)	4.44 (0.63)	4.73 (0.46)	4.50 (0.53)
	Taught skills that can be implemented in practice	3.82 (0.4)	4.19 (0.66)	4.67 (0.49)	4.75 (0.46)
	Recommend to friend/colleague	3.4 (1.07)	3.94 (0.77)	4.6 (0.51)	4.38 (0.52)
	Taught skills that enhance ability to educate patients to manage cancer & treatment	3.64 (1.03)	4.19 (0.66)	4.73 (0.46)	4.75 (0.46)
Satisfaction, µ (σ)	Overall course	3.44 (1.24)	4.06 (0.77)	4.6 (0.51)	4.63 (0.52)
	Location	4.73 (0.65)	3.50 (1.03)	4.27 (0.88)	4.13 (0.99)
	Time spent/duration of course	3.4 (1.43)	3.94 (0.93)	4.53 (0.52)	4.25 (0.46)
	Teaching methods used:				
	Reading materials	3.64 (1.12)	4.00 (0.82)	4.53 (0.52)	4.5 (0.53)
	Learning activities	3.64 (1.12)	4.19 (0.66)	4.53 (0.52)	4.5 (0.53)
	Opportunities for skills practice	3.7 (1.16)	4.19 (0.75)	4.53 (0.52)	4.5 (0.53)
	Opportunities to exchange ideas	4.09 (0.54)	4.44 (0.73)	4.6 (0.51)	4.5 (0.53)
	Opportunities to build collegial relationships	3.9 (0.57)	3.94 (0.85)	4.73 (0.46)	4 (0.53)
Facilitator, µ (σ)	Ability to foster a safe, supportive learning environment	4.36 (0.5)	4.19 (0.66)	4.87 (0.35)	4.75 (0.46)
	Ability to provide helpful feedback	3.91 (0.83)	4.25 (0.58)	4.73 (0.46)	4.75 (0.46)
	Ability to model skills taught while interacting	3.82 (0.75)	4.00 (0.82)	4.80 (0.41)	4.75 (0.46)
	Knowledge of content	4.09 (0.83)	4.19 (0.66)	4.8 (0.41)	4.63 (0.52)
	Clarity of presentation	3.50 (1.27)	4.13 (0.72)	4.73 (0.46)	4.63 (0.52)

µ = mean; σ = standard deviation.

## Data Availability

Data can be made available upon reasonable request to the corresponding author and available in a de-identified format.

## Supplementary Materials

The following supporting information can be downloaded at: https://www.mdpi.com/article/10.3390/cancers15061811/s1, Supplement S1. The 5As tip sheet for immunotherapy treatment toxicities (adapted from Refs. [26,39,40,41,42]); Supplement S2. Cancer coaching skills checklist for fidelity; Supplement S3. Survey of nurses’ confidence in self-management support skills.
