# Supplementary material for: Impact of a Training Program on Oncology Nurses’ Confidence in the Provision of Self-Management Support and 5As Behavioral Counseling Skills
Source: Cancers (Basel). 2023 Mar 16;15(6):1811. doi: 10.3390/cancers15061811 (PMC10046590; doi:10.3390/cancers15061811)
Supplement: Supplementary file 1 [file cancers-15-01811-s001.zip › cancers-2145129-supplementary.pdf]

Supplement S1. Application of 5As to Support Patient Self-Management of Immunotherapy Treatment Toxicities

5As Step	Tips and Strategies
**ASSESS**	
**Establish Rapport with patient and family**<br>• Active listening, trust, respect, genuineness, empathy<br>• Assess/respond to patient concerns, feelings, distress	• Use open ended questions that invite more than a yes or no response (e.g., “Tell me more about….”)<br>• Avoid confrontational “why” questions<br>• Restate (reflect back) what you have heard (e.g., “It sounds like….”)<br>• Use good eye contact and non-verbal cues (e.g., nodding) to indicate you are listening<br>• Acknowledge/normalize their emotions (e.g., “So you are feeling….”, “It is not uncommon…to have these feeling…”)
**Establish a mutually agreed upon agenda for the visit**<br>• Draw on pre-visit assessments (e.g., ESAS)<br>• Identify the patient’s priority concerns	• Assess/clarify the patient’s expectations at each visit (e.g., I have some things to ask you about, but first, tell me what you want to focus on during today’s visit?”)<br>• I see that your pain score is higher this week. Could we talk about this today?
**Assess patient risk factors for poor health**<br>(e.g., performance status, co-morbidities, mental health conditions, coping, medications, social support, financial issues, education)	• Understanding of cancer and what it means to them. (e.g., “How has having cancer impacted on your life? What worries you the most?”)<br>• Understanding of genomic/biomolecular tests, prognosis, treatment options including I-O therapies (e.g., What do you think about the I-O therapy your doctor has recommended?)<br>• Disease and treatment-related symptoms, psychological distress, coping<br>• How have they managed previous health issues? What activities do they use to manage their health/cancer situation?<br>• Barriers to self-management (e.g., What difficulties have you experienced in managing….?)
**Assess patient’s readiness to change to take on new self-management behaviors**<br>(e.g., symptom monitoring, medication management, exercise)	• From your perspective, what is the most important aspect of your health that you want to maintain or improve while on I-O treatment?<br>• On a scale of 0 to 10 how confident are you that you can make this change? Why this number and not a lower number?<br>• What would help you feel more confident?<br>• Explore the pros/cons of taking action
**Encourage patients to use health risk appraisal tools**	• e.g., ESAS. model the use of the tools and discuss the results
**ADVISE**	
**Use behavioral, psychosocial, and tailoring strategies to provide self-management education**	• Involve the patient in their own care. Ask their permission to share information/advice. (e.g., I have some information here on strategies for how to….Would this be helpful for you?”<br>• Tailor strategies/messages to patient age, culture, ethnicity, and health literacy<br>• Use adult learning principles and active learning techniques<br>• Give clear, specific information and advise about health behaviors that is personalized to the patient, including potential risks/harms and benefits of recommended strategies<br>• Teach skills patients need to manage their health<br>• Assist patients to acknowledge/understand their emotional responses, feelings, attitudes related to cancer<br>• Build patient self-efficacy by indicating your confidence that they can take on new skills/behaviors and acknowledging their personal strengths and previous successes in managing their health

5As Approach - Tip Sheet for I-O Care

5As Step	Tips and Strategies
**ADVISE**	
**Use Ask-Tell-Ask to make sure patient receives the information**	• Ask: Tailor information to the patient by asking what they know and what they want to know<br>• Tell: Share information the patient wants to know in simple concise language<br>• Ask: Have the patient repeat back what they heard in their own words. Do they have questions or require more information.
**Use the Close-the-Loop technique to assess patient understanding of the information**	• Ask the patient to summarize their understanding of the key points from the discussion.<br>• Clarify and reinforce information that was not understood
**Assist patients to use information from self-monitor tools (e.g., pain scores, temperature)**	• Review and discuss how to use tools, interpret the information, and how to respond
**Encourage patients to use monitoring methods (e.g., journals, diaries, logs, health records)**	• e.g., A Self-Monitoring Guide for Patients Receiving Checkpoint Inhibitors. Nova Scotia Health. http://www.nshealth.ca/sites/nshealth.ca/files/patientinformation/nsccp1673.pdf<br>• Cancer organizations for patients often have excellent self-management support tools using these methods
**AGREE**	
**Establish goals for improving health at different stages of the cancer continuum**	• Respect patient autonomy to make choices about improving their health<br>• Encourage shared-decision-making and the patient's involvement in determining the priority areas of focus for improving their health<br>• The goal should be very important to the patient
**Develop an action plan to achieve goals**	• Help the patient set realistic expectations and incorporate discussion about the patient's values and preferences in the decision-making process<br>• Utilize strategize above to consider patient's readiness and barriers to change<br>• Patients should feel confident (e.g., 7/10 on a confidence scale) that they can achieve the plan.<br>• Be specific. Determine what (specific action), how much (time, distance, amount), when (time, days of the week), how often.<br>• Use Close-the-Loop to ensure the patient understands the action plan and any instructions.
**Monitor progress in goal achievement**	• Ask how well the strategies worked and explore barriers to achievement

5As Approach - Tip Sheet for I-O Care

5As Step	Tips and Strategies
**ASSIST**	
• Assist the patient to explore their motivation to improve their health and discuss potential barriers to implementing the action	• See assessment of readiness above
• Assist the patient to identify problems in achieving health goals and generate solutions	• What gets in the way of managing their side effects? What might help overcome this barrier?<br>• Would you like to hear some suggestions from me?<br>• Out of all the strategies we have discussed today, which one do you think will work best for you?
• Link the patient to relevant cancer centre and community services and providers	• Consider cancer specific and non-cancer specific resources (e.g., supportive care cancer services, support groups, fitness clubs, dieticians, health coaches, online information)
**ARRANGE**	
• Arrange follow-up appointments by telephone, email, or additional or regular cancer clinic visits	• Plan follow-up based on patient's needs, preferences, and availability<br>• Determine the focus of follow-up appointments (e.g., assessment, monitoring, coaching, reinforcement)<br>• Facilitate health system navigation, coordination of care, and referrals to specialist providers and self-management support services and resources

Adapted from:

Glasgow, R.E., Funnell, M.M., Bonomi, A.E., Davis, C., Beckham, V., & Wagner, E.H. (2002). Self-Management Aspects of Improving Chronic Illness Care Breakthough Series: Implementation with Diabetes and Heart Failure Teams. Annals of Behavioral Medicine, 24(2), 80-87.

Medland, M. (2009). Self-Management Support: A Study and Implementation Guide for Health Care Professionals. Fraser Health Authority, BC: Fraser Health.

Registered Nurses Association of Ontario. (2010). *Strategies to Support Self-Management in Chronic Conditions: Collaboration with Clients*. Toronto, ON. RNAO

South West Self-Management Program. (2018). *Self-Management Toolkit. A Resource for Health Care Providers*. www.self-management.ca

Whitelock, Orleans, Pender, & Allan. (2002). Evaluating Primary Care Behavioral Counselling Interventions: An Evidence-Based Approach. *American Journal of Preventive Medicine*, 22(4), 267-284).

Supplement S2. Cancer Coaching Skills Checklist for Fidelity

**Cancer Centre:** ☐ Centre 3 ☐ Centre 2 ☐ Centre 1	
**Date of Assessment (DD/MMM/YYYY):**	
Preparedness of the health coach *(ask these <u>ideally</u> before the visit with the participant)*	
	Coach is familiar with the content of the I-Can-Manage Cancer modules
	Coach can describe open-ended questions that will be used to establish rapport
	Coach can describe participant's most recent action plan
	Coach can name his/her goals for the visit
Comments:	
Greeting and initial communication	
	Coach gives the participant a professional greeting
	Coach establishes rapport.
Comments:	
**Assess:** Setting the agenda for the coaching visit	
	Coach establishes collaborative agenda visit with participant.
	Coach restates and documents what participant states (reflections)
	Coach asks if participant has any more questions until the participant has no more to say
	Coach asks patient if okay to talk about some priority issues (e.g. safe management of treatment side-effects/cancer symptoms, use of medications)
	Coach assesses readiness (importance and confidence)
	Coach asks participant to prioritize discussion topics and select two or three for the session
	Coach and participant agree on the agenda for the visit
Comments:	
**Advise:** Ask-Tell-Ask and Teach Back	
	Coach actively listens without interrupting
	Coach's comments and tone are used in a friendly, accessible and not judgmental manner
	Coach asks participant questions relevant to the topic at hand
	Coach provides information or advice only when participant asks or doesn't know
	treatment effects
	If coach does not know information, says "How can we find that information?" or "I will find out and get back to you"
	Coach teaches the patient steps in problem-solving and patient comes up with way to get the information
	Coach teaches the patient about self-monitoring and adjusting behaviours/actions using this data

	Coach ensures the participant understands the information (in their own words)-asks patient to talk back advise given and specific actions to be taken
	Coach takes advantage of learning moments to ask questions (e.g. What is your goal for managing pain?)
Comments:	

Supplement S3: Pre/Post Training: Nurses' Confidence in SMS Micro Skills

Items	Strongly Disagree	Disagree	Neither Agree/ Disagree	Agree	Strongly Agree
**1. Overall the course…….**					
a. Was a valuable use of my time	1	2	3	4	5
b. Was relevant to my professional practice	1	2	3	4	5
c. Taught skills that I can implement in my practice	1	2	3	4	5
d. Is something that I can recommend to a friend or colleague	1	2	3	4	5
e. Taught skills that will enhance how I educate patients to manage cancer and treatment	1	2	3	4	5
**2. Please rate your satisfaction with the……..**	**Very Satisfied**		**Neither**		**Very Dissatisfied**
a. Overall course	1	2	3	4	5
b. Location	1	2	3	4	5
c. Time spent/duration of the course	1	2	3	4	5
d. Teaching methods used					
• Reading materials	1	2	3	4	5
• Learning activities	1	2	3	4	5
• Opportunities for skills practice	1	2	3	4	5
e. Opportunities to exchange ideas	1	2	3	4	5
f. Opportunities to build collegial relationships	1	2	3	4	5
**3. Please rate each facilitator in terms of his or her……..**					
a. Ability to foster a safe, supportive learni environment	1	2	3	4	5
b. Ability to provide helpful feedback	1	2	3	4	5
c. Ability to model the skills taught while interacting with you today	1	2	3	4	5
d. Knowledge of content	1	2	3	4	5
e. Clarity of presentation	1	2	3	4	5

4. As a result of this course, how confident are you in the following areas (1- no confidence to 5-very confident):

	No Confidence		Somewhat Confident		Very Confident
a. Defining self-management support and how it differs from patient education	1	2	3	4	5
b. Supporting patients in making difficult decisions	1	2	3	4	5

c. As a result of this course, how confident are you in using the following elements of the 5A's Model to provide patients with self-management support

	No Confidence		Somewhat Confident		Very Confident
• **Assess**					
▪ Establish rapport	1	2	3	4	5
▪ Setting the visit agenda	1	2	3	4	5
▪ Assess client readiness to make a change	1	2	3	4	5
▪ Goal setting	1	2	3	4	5
• **Advise**					
▪ Tailoring strategies for selection	1	2	3	4	5
▪ Ask-tell-ask	1	2	3	4	5
▪ Closing the loop	1	2	3	4	5
• **Agree**					
▪ Action plans and follow-up	1	2	3	4	5
• **Assist**					
▪ Problem solving	1	2	3	4	5
▪ Linking to community resources	1	2	3	4	5
▪ Teaching self-monitoring skills	1	2	3	4	5
▪ Review goal and action plan	1	2	3	4	5
• **Arrange**					
▪ Follow-up	1	2	3	4	5
